# PdPbAg alloy NPs immobilized on reduced graphene oxide/In_2_O_3_ composites as highly active electrocatalysts for direct ethylene glycol fuel cells

**DOI:** 10.1039/d2ra03248a

**Published:** 2022-07-07

**Authors:** Zhirui Wu, Yuting Zhong, Zhiguo Wang, Ling Li, Xiaoguang Liu

**Affiliations:** School of Chemistry and Chemical Engineering, Hubei University Hubei Wuhan 430000 P. R. China wzg96513@hubu.edu.cn liling402431@hotmail.com liuxiaoguang402@hotmail.com

## Abstract

rGO-modified indium oxide (In_2_O_3_) anchored PdPbAg nanoalloy composites (PdPbAg@rGO/In_2_O_3_) were prepared by a facile hydrothermal, annealing and reduction method. Electrochemical tests showed that the as-prepared trimetallic catalyst exhibited excellent electrocatalytic activity and high resistance to CO poisoning compared with commercial Pd/C, mono-Pd and different bimetallic catalysts. Specifically, PdPbAg@rGO/In_2_O_3_ has the highest forward peak current density of 213.89 mA cm^−2^, which is 7.89 times that of Pd/C (27.07 mA cm^−2^). After 3600 s chronoamperometry (CA) test, the retained current density of PdPbAg@rGO/In_2_O_3_ reaches 78.15% of the initial value. Its excellent electrocatalytic oxidation performance is attributed to the support with large specific surface area and the strong synergistic effect of PdPbAg nanoalloys, which provide a large number of interfaces and achievable reactive sites. In addition, the introduction of rGO into the In_2_O_3_ matrix contributes to its excellent electron transfer and large specific surface area, which is beneficial to improving the catalytic ability of the catalyst. The study of this novel composite material provides a conceptual and applicable route for the development of advanced high electrochemical performance Pd-based electrocatalysts for direct ethylene glycol fuel cells.

## Abstract 2

rGO-modified indium oxide (In_2_O_3_) anchored PdPbAg nanoalloy composites (PdPbAg@rGO/In_2_O_3_) were prepared by a facile hydrothermal, annealing and reduction method.

## Graphical abstract

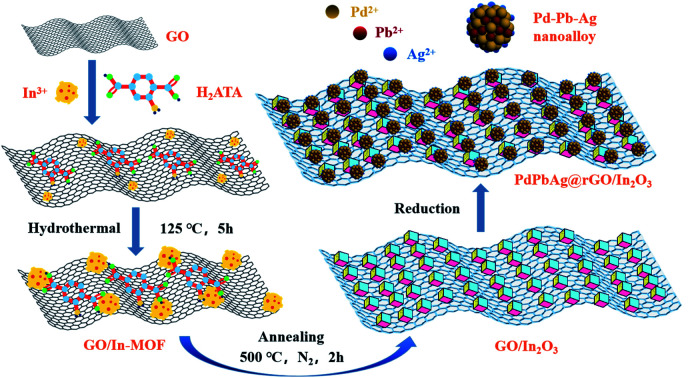

## Introduction

Modern society urgently needs to replace traditional fossil fuels with new clean energy sources.^1^ Direct alcohol fuel cells (DAFCs) are currently considered attractive candidates for reducing future energy demand.^2^ Direct ethylene glycol fuel cells (DEGFCs) have unique advantages such as safe storage, easy availability, high energy density, and convenient transportation.^3^ However, the anodic electrocatalysts of DAFCs tend to adsorb the toxic intermediates generated in the anodic ethylene glycol oxidation reaction, resulting in decreased electrocatalytic activity and stability.^4^ The design of anode electrocatalysts usually depends on the size, surface electronic structure, shape, and composition of the material.^5,6^

It is effective to further construct porous structures with large surface area and active sites. Metal–organic frameworks (MOFs) have the advantages of high porosity, tunable pore size, and large internal specific surface area.^7,8^ Reduced graphene oxide (rGO) can anchor metal nanoparticles through its inherent residual oxygen-containing functional groups and defect sites,^9^ which can enhance the conductivity of catalysts. Improve the dispersion of the supported metals to enhance the electrocatalytic activity and stability of EGOR by providing more active centers and electron transport.^10,11^ MOFs are often used as ideal templates for the preparation of MOFs-derived porous metal oxides with high catalytic activity and stability to compensate for the poor electrical conductivity and acid-base corrosion resistance of MOFs. In_2_O_3_ is a wide bandgap n-type semiconductor that has been widely used in the microelectronic field and gas sensors.^12,13^ Previous studies have shown that it has good catalytic activity and low resistivity.^14^ However, there are few reports on the application of In_2_O_3_ used as the substrate in the EGOR. Pd-based materials have abundant storage and good catalytic activity, so they are often used as effective active components in anodic alcohol oxidation reaction (AOR).^15,16^ However, many studies have shown that single Pd catalysts have poor electrocatalytic performance and stability. Therefore, the d-band center of Pd can be effectively tuned by modifying the Pd surface by doping with appropriate transition metals or alloying Pd.^16^ In addition, metal particles supported on supports can promote the uniform dispersion of nanoparticles (NPs). For example, Pd-based bimetallic catalysts Pd-Cu^17^ and Pd–Sn/Pd–Ni/CNT;^18^ trimetallic catalysts PdCuBi/C,^19^ Pd–Co–Ni/G^20^ and Pd–Ru–Bi.^21^ Thereby increasing the active center of electrocatalyst, promoting the transfer of electrons, and optimizing the performance of the catalyst.^22,23^ At the same time, it exhibits excellent synergistic effect and high resistance to CO poisoning, leading to improved electrocatalytic performance of Pd catalysts.^15^

**Scheme 1:**
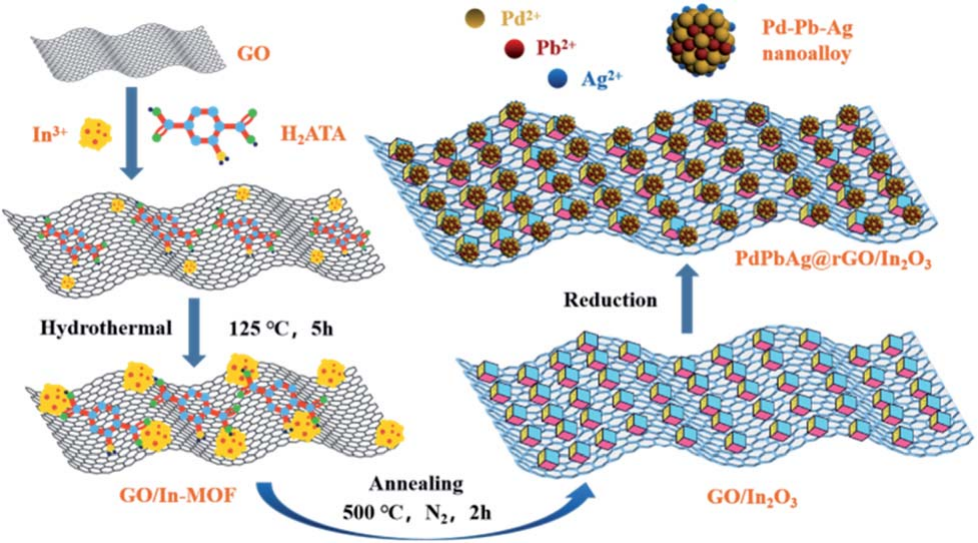
Schematic diagram of the preparation process of PdPbAg@rGO/In_2_O_3_ electrocatalyst.

Taking the above considerations into account, in this study, graphene oxide-modified In_2_O_3_ nanoparticles were successfully synthesize by hydrothermal method and annealing at 500 °C under N_2_ atmosphere. The PdPbAg NPs were then embedded into the previously obtained GO/In-MOF, and the reduction method was used to obtain the PdPbAg@rGO/In_2_O_3_ trimetallic electrocatalyst, as shown in Scheme 1. Compared with Pd/C, the current density of PdPbAg@rGO/In_2_O_3_ (213.89 mA cm^−2^) for EG is 7.89 times higher than that of Pd/C (27.1 mA cm^−2^). At the same time, PdPbAg@rGO/In_2_O_3_ has the highest catalytic activity, the lowest *E*_onset_, the smallest resistance to EG, and better anti-toxicity. After 3600 s CA test, the retained current density of PdPbAg@rGO/In_2_O_3_ still maintains the highest value of 64.79 mA cm^−2^ and 78.15% of the original current density, which is superior to that of Pd/C (4.03 mA cm^−2^, 20.15%). This is attributed to the strong electronic effect between Pd–Pb–Ag nanoalloys, which can significantly enhance the adsorption of oxygen-containing species on the Pd surface. Thereby accelerating the oxidation of intermediate carbon species on the Pd surface and the removal of adsorbed CO species. Meanwhile, the carrier rGO/In_2_O_3_ enhances the specific surface area and electron transfer rate, with good electrical conductivity and unique structure, providing abundant active sites for the Pd–Pb–Ag alloy NPs.

## Experimental

### Materials and reagents

Graphite powder was purchased from Sigma-Aldrich. Indium nitrate (In(NO_3_)_3_·xH_2_O, 99.9%) and 2-Aminoterephthalic acid (H_2_ATA, H_2_NC_6_H_3_-1,4-(CO_2_H)_2_, 98%), palladium(ii) chloride (PdCl_2_, 60%) were purchased from Shanghai Macklin Co. Ltd., lead nitrate (Pb(NO_3_)_2_, 99%), silver nitrate (AgNO_3_, 99.8%), sodium borohydride (NaBH_4_, 98%), concentrated sulfuric acid (H_2_SO_4_, 98%), hydrogen peroxide (H_2_O_2_, 30%), hydrochloric acid (HCl, 37.0%), potassium permanganate (KMnO_4_, 99%), sodium nitrate (NaNO_3_, 99%), potassium hydroxide (KOH, 85%), ethanol (CH_3_CH_2_OH, 99.7%), ethylene glycol (EG, 99.5%), isopropyl alcohol (CH_3_CHOHCH_3_, 99.7%), *N*,*N*-Dimethylformamide (DMF, 99.5%) were purchased from Sinopharm chemical reagent Co. Ltd. Nafion (5%) was purchased from Aladdin. All reagents were analytical pure (AR) and directly used in this experiment.

### Synthesis of GO/In_2_O_3_

Graphene oxide (GO) was prepared from graphite powder *via* the modified Hummer's method.^24,25^ 1.4670 g of In(NO_3_)_3_·*x*H_2_O, 0.2337 g of H_2_ATA, 12.4 mL of DMF and 0.0467 g of GO were added to the beaker and ultrasonicated for 30 min. The mixture was then transferred into a 50 mL teflon-lined stainless steel autoclave and heated at 125 °C for 5 h. After cooling to room temperature, the obtained black product was filtered, washed three times alternately with deionized water, DMF and absolute ethanol, and dried under vacuum at 40 °C overnight. Finally, the obtained product was annealed at 500 °C for 2 h under N_2_ atmosphere to obtain the precursor GO/In_2_O_3_.

### Synthesis of PdPbAg@rGO/In_2_O_3_ electrocatalysts

50 mg of GO/In_2_O_3_ was dispersed in EG (50 mL) by ultrasonication, and then 5 mL PdCl_2_ (0.0189 M) was added to the above solution and stirred evenly. After that, 250 μL of Pb(NO_3_)_2_ (0.0756 M) and 1890 μL of AgNO_3_ (0.01 M) were injected into the above solution under magnetic stirring. And this mixture was stirred for 3 h at room temperature. Then 80 mg of NaBH_4_ was dissolved in 20 mL of deionized water and slowly added dropwise into the mixture solution under vigorous stirring for 4 h. The product was collected by filtration, washed three times alternately with deionized water and ethanol, and finally dried under vacuum at 40 °C overnight to get PdPbAg@rGO/In_2_O_3_. Using the same procedure, the PdPb@rGO/In_2_O_3_, PdAg@rGO/In_2_O_3_ and Pd@rGO/In_2_O_3_ electrocatalysts were also synthesized without the addition of AgNO_3_, Pb(NO_3_)_2_.

### Physical characterization

The crystalline structures of prepared electrocatalysts were characterized by X-ray diffraction (XRD) using a Bruker-D8 Advance X-ray diffractometer equipped with Cu Kα radiation (*λ* = 1.5406 Å). The XRD patterns were collected at 2θ values ranging from 5 to 80°. The morphology and chemical component of catalyst were investigated by field emission scanning electron microscopy (FE-SEM, Zeiss ULTRA 55) and energy-dispersive X-ray spectrometer (EDS). The particle size distribution and microstructure of composites were investigated using a high-resolution transmission electron microscope (HRTEM, FEI Tecnai F20). The chemical valence state of element was determined by X-ray photoelectron spectroscopy (XPS, Thermo ESCALAB 250Xi) with a monochromatic Al Kα source of 1486.6 eV. The inductively-coupled plasma optical emission spectrometer (ICP-OES) measurements were conducted on Agilent ICP-OES 725 analyzer.

### Electrochemical measurements

All the electrochemical measurements were operated in CHI660E electrochemical workstation (CH Instruments, Inc., Shanghai) with standard three-electrode system. The glassy carbon electrode (GCE, 0.1256 cm^2^), Pt wire and Hg/HgO electrode were used as the working, counter and reference electrode, respectively. In a typical procedure, 5 mg of catalyst and 5 μL of 0.5 wt% Nafion solution were mixed with 950 μL of isopropanol, followed by sonication for 30 min to produce a uniformly dispersed suspension, and then 10 μL of the suspension solution containing catalyst was dropped onto the surface of the GCE and dried at room temperature to obtain the working electrode. First, the working electrode was tested by cyclic voltammetry (CV) in the range of - 0.9 V to 0.8 V at a scan rate of 50 mV s^−1^ in 1.0 M KOH solution to activate the prepared catalyst. The electrocatalytic oxidation activity of ethylene glycol was characterized by CV in N_2_-treated solution of 1.0 M KOH + 0.5 M EG. The scanning potential range was from - 0.9 V to 0.8 V and the scan rate was 50 mV s^−1^. Chronoamperometry (CA) experiments were performed at a potential of - 0.1 V for 3600 s to test the durability of the catalysts. Electrochemical impedance spectroscopy (EIS) measurements were implemented on different catalysts at open circuit potentials from 0.01 to 10^6^ Hz. Before testing, ultrapure N_2_ was introduced into the electrolyte for 30 min to remove dissolved oxygen.

## Results and discussion

### Physical characterization of catalysts

**Fig. 1:**
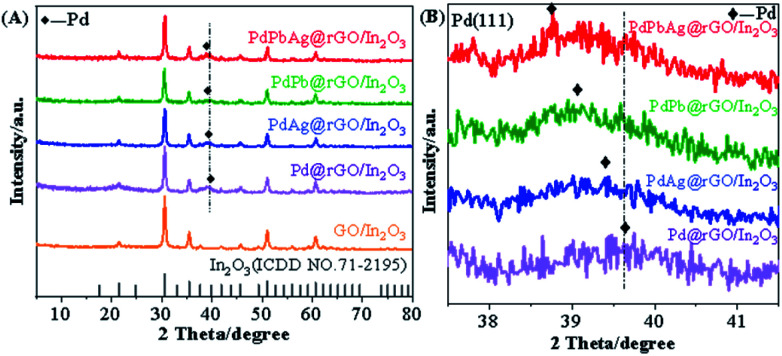
(A) XRD patterns of PdAgPb@rGO/In_2_O_3_, PdPb@rGO/In_2_O_3_, PdAg@rGO/In_2_O_3_, Pd@rGO/In_2_O_3_, GO/In_2_O_3_ and In_2_O_3_, (B) diffraction peaks of Pd in PdAgPb@rGO/In_2_O_3_, PdPb@rGO/In_2_O_3_, PdAg@rGO/In_2_O_3_ and Pd@rGO/In_2_O_3_.

The crystal structures of all samples were determined by XRD. The corresponding patterns of PdAgPb@rGO/In_2_O_3_, PdPb@rGO/In_2_O_3_, PdAg@rGO/In_2_O_3_, Pd@rGO/In_2_O_3_, GO/In_2_O_3_ and In_2_O_3_ are shown in Fig. 1. It can be seen that the main diffraction peaks of the support GO/In_2_O_3_ at 2*θ* = 21.60°, 30.54°, 35.46°, 41.74°, 45.58°, 51.00°, 60.71° correspond to the (211), (222), (400), (332), (134), (440), (622) of the standard card of In_2_O_3_ (ICDD NO. 71-2195), indicating the successful synthesis of In_2_O_3_.^26,27^ At 2*θ* = 25.0°, the peak of In_2_O_3_ overlap with the broad peaks of graphene, making the (002) plane of graphene unobservable.^28^ In addition, the diffraction peaks around 39.8°, for the four catalysts can be attributed to (111) crystal planes of face-centered cubic (fcc) crystalline of Pd.^25^ In addition, the Pd (111) facets of PdPbAg@rGO/In_2_O_3_, PdPb@rGO/In_2_O_3_, and PdAg@rGO/In_2_O_3_ are shifted compared with the diffraction angles of Pd@rGO/In_2_O_3_ (enlarged XRD pattern in Fig. 1(B)). The Pd (111) planes are shifted to 38.8°, 39.1° and 39.4° from 39.6°, respectively.^29^ This indicates that alloys are formed between Pd, Pb and Ag, which changes the lattice constant of Pd and increases the synergistic effects and electrocatalytic activity of Pd–Pb–Ag for EGOR. Furthermore, in bimetallic PdPb, PdAg and trimetallic PdPbAg@rGO/In_2_O_3_ catalysts, due to the low Pb and Ag content on the catalyst surface, or thin and amorphous phase, no obvious Pb and Ag diffraction peaks were observed.^30,31^ From the above analysis, it can be seen that the formation of metal nanoalloys is of great significance for improve the synergistic effect and catalytic activity of ethylene glycol electrooxidation.

**Fig. 2:**
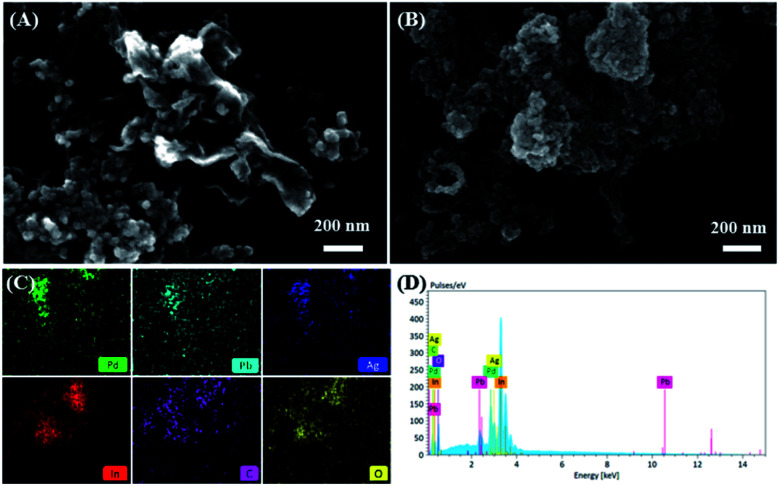
FE-SEM images of (A) GO/In_2_O_3_, (B) PdPbAg@rGO/In_2_O_3_, (C) HAAD-SSEM elemental mapping of Pd, Pb, Ag, In, C, O, (D) EDS of PdPbAg@rGO/In_2_O_3_.

The surface morphologies of PdPbAg@rGO/In_2_O_3_ were investigated by FE-SEM. Comparing the FE-SEM image of GO/In_2_O_3_ (Fig. 2(A)), Fig. 2(B) shows the FE-SEM image of the PdPbAg@rGO/In_2_O_3_ catalyst, the nanoparticles of PdPbAg are uniformly loaded to the support. By analyzing the element mapping diagram (Fig. 2(C)), the catalyst contains Pd, Pb, Ag, In, C and O elements, confirming the uniform dispersion of Pd–Pb–Ag nanoalloys. Additionally, in Fig. 2(D), the EDS spectrum further shows the presence of In, Pd, Ag Pb, C and O. The actual atomic loadings of Pd, Pb, and Ag in the PdPbAg@rGO/In_2_O_3_ catalyst are 13.23%, 5.34%, and 3.36%, respectively. The mass percentages of Pd, Pb and Ag measured by ICP-OES are 12.45%, 6.02% and 4.25%, respectively. The test results of the two are close, which are also consistent with the initial addition ratio of the reactants. It demonstrated that the Pd, Pb and Ag precursors have been completely converted into products, and the trimetallic composite was successfully synthesized.^32^

**Fig. 3:**
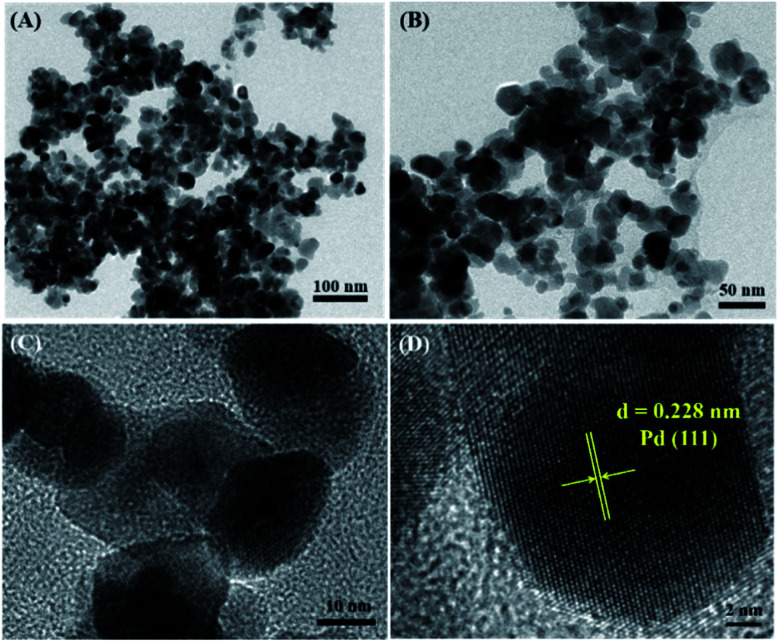
(A), (B), (C) and (D) HR-TEM images of PdPbAg@rGO/In_2_O_3_ at different magnifications.

The morphology and particle size distribution of PdPbAg@rGO/In_2_O_3_ were further analyzed with HR-TEM (Fig. 3). Fig. 3(D) show the crystalline properties of PdPbAg@rGO/In_2_O_3_. The well-shaped lattice fringes wuth interplanar spacing of 0.228 nm, corresponding to the Pd (111) plane. Compared with the pure Pd (0.223 nm), the interplanar spacing of Pd in the catalyst is slightly larger. This indicates that the lattice of Pd expands with the addition of Pb and Ag NPs, confirming the formation of PdPbAg nanoalloys.^33^ This small PdPbAg nanoalloy can easily obtain more active cites, which is beneficial to the electrocatalytic oxidation of ethylene glycol.

**Fig. 4:**
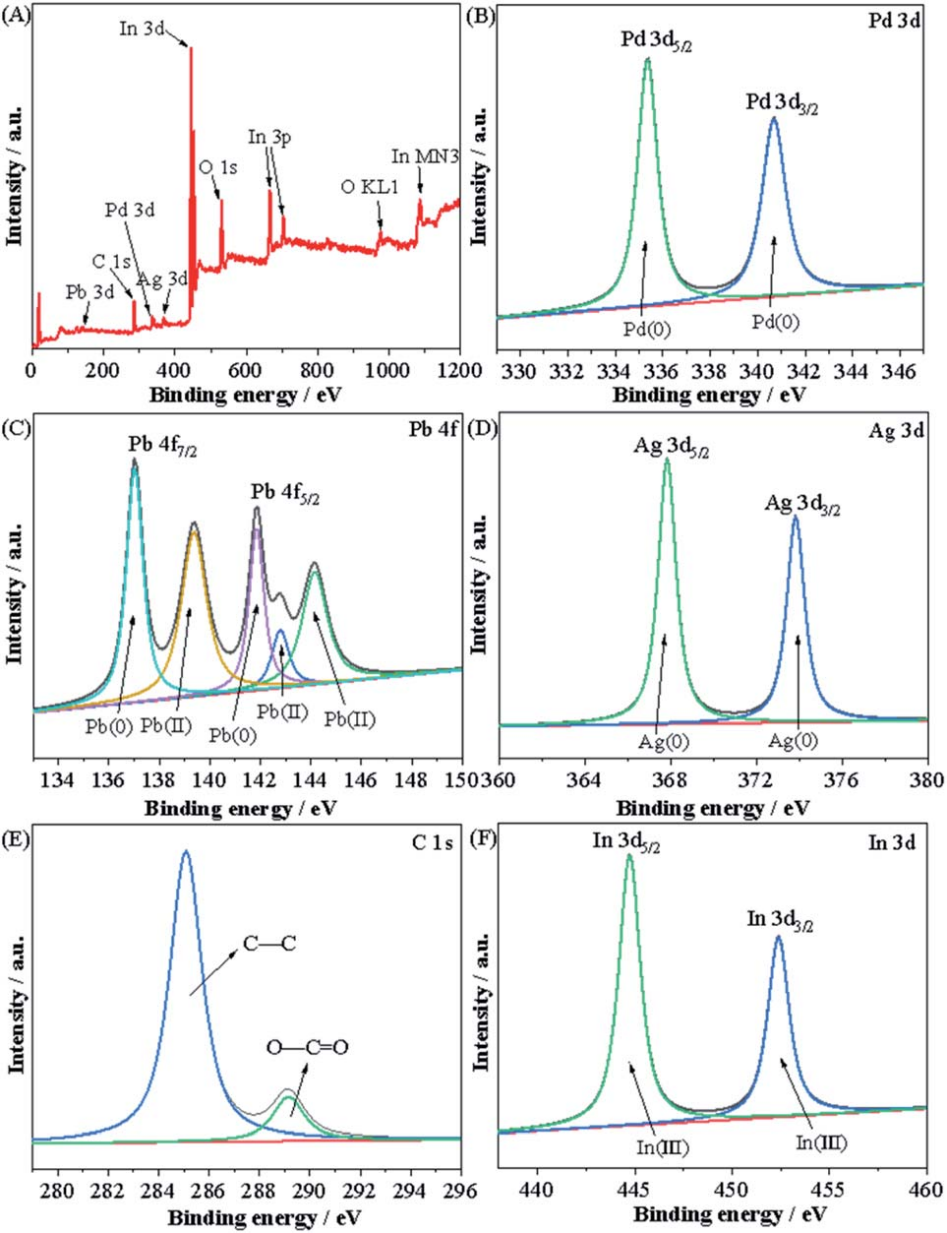
XPS spectra of (A) Survey, (B) Pd 3d, (C) Pb 4f, (D) Ag 3d, (E) C 1 s and (F) In 3d in PdPbAg@rGO/In_2_O_3_.

Fig. 4 shows the XPS spectrum of PdPbAg@rGO/In_2_O_3_. According to the XPS spectrum, the elemental valence states of Pd, Pb and Ag in the catalyst can be determined. The survey of Fig. 4(A) shows the photoelectron peaks of Pd, Pb, Ag, In, C and O in the trimetallic catalyst. As shown in Fig. 4(B), the two typical peaks at 335.3 eV and 340.7 eV are assigned to Pd 3d_5/2_ and 3d_3/2_ of Pd(0). There are no obvious other valence peaks, which means that Pd ions are completely reduced to Pd(0). The binding energies (BE) of Pb 4f were depicted in Fig. 4(C), the two peaks at 137.0 eV and 141.8 eV are ascribed to Pb(0) 4f_7/2_ and 4f_5/2_, while the other two peaks at 142.8 eV and 144.2 eV correspond to Pb(ii) 4f_7/2_ and Pb(ii) 4f_5/2_, such as Pb–O.^34,35^ The surface modification of Pb/PbO NPs can enhance the electrocatalytic performance for EG.^36,37^Fig. 4(D) shows the XPS of Ag 3d, and the peaks at BE of 368.0 eV and 378.8 eV correspond to Ag 3d_5/2_ and Ag 3d_3/2_, respectively, which is the characteristic peak of Ag(0). In addition, the BE of Pd 3d, Pb 4f and Ag 3d in PdPbAg@rGO/In_2_O_3_ show a slight negative displacement compared to pure Pd (Pd 3d_5/2_ = 335.0 eV, Pd 3d_3/2_ = 340.3 eV), Pb (Pb 4f_7/2_ = 136.9 eV, Pb 4f_5/2_ = 141.7 eV) and Ag (Ag 3d_5/2_ = 367.7 eV, Ag 3d_3/2_ = 374.2 eV), indicating the formation of PdPbAg nanoalloys. Fig. 4(E) is the XPS spectrum of C 1 s, two peaks can be seen in the figure, the prominent peak at 285.1 eV belongs to the C–C bond of rGO, and the other peak at 289.1 eV corresponds to the O–C=O functional group of rGO.^38^ It can be found that the peaks of oxygen-containing functional groups are particularly small, indicating that GO is reduced to rGO in the electrocatalyst. Fig. 4(F) depicts the XPS spectrum of In 3d showing two peaks at 444.8 and 452.4 eV, which can be attributed to the characteristic spin–orbit splitting 3d_5/2_ and 3d_3/2_, respectively. This proves that the valence state of indium in In_2_O_3_ is mainly + 3.^39^ The BE of In also shows a slight negative shift compared with pure In (In 3d_5/2_ = 443.8 eV, In 3d_3/2_ = 451.4 eV), indicating there is an interaction between In_2_O_3_ and rGO or metals.

### Electrochemical study on electrooxidation of ethylene glycol

**Fig. 5:**
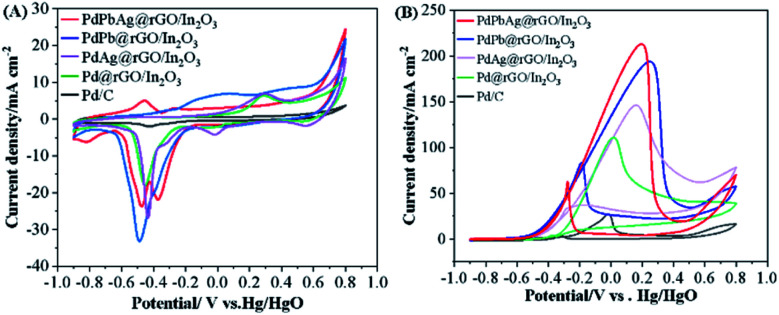
CV curves of the electrocatalysts in (A) 1 M KOH and (B) 1 M KOH + 0.5 M EG (scan rate: 50 mV s^−1^).

The typical cyclic voltammetry (CV) curves of commercial PdPbAg@rGO/In_2_O_3_, PdPb@rGO/In_2_O_3_, PdAg@rGO/In_2_O_3_, Pd@rGO/In_2_O_3_ and Pd/C, were recorded in N_2_-saturated 1.0 M KOH at the scanning rate of 50 mV s^−1^, as shown in Fig. 5(A). It can be observed that the oxidation peak of each catalyst is around 0.3 V and the reduction peak is around - 0.5 V. The electrochemical active surface area (ECSA) of a catalyst is closely related to the active center of the catalyst and can be calculated by the following formula:
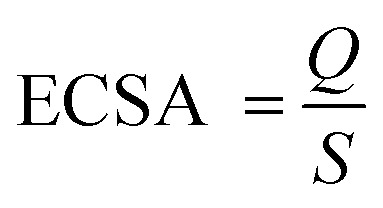


*Q* in the formula is the coulombic charge observed during the reduction of Pd oxide,^40^ and *S* is the proportionality constant during the reduction of the PdO monolayer, which is 0.405 mC cm^−2^.^17,21^ Compared with Pd/C (6.95 cm^2^), Pd@rGO/In_2_O_3_ (37.12 cm^2^), PdAg@rGO/In_2_O_3_ (40.39 cm^2^) and PdPb@rGO/In_2_O_3_ (43.47 cm^2^), PdPbAg@rGO/In_2_O_3_ (47.39 cm^2^) has the highest ECSA values (Table 1). It is pointed out that there may be highly active reaction centers in ternary alloy catalysts. This is related to the wide extended surface area of the support In_2_O_3_ and the high conductivity of rGO.

**Table 1:** The results of CV measurements of all catalysts modified electrodes. (a: in 1.0 M KOH, b: in 1.0 M KOH + 0.5 M EG)

Samples	ECSA^*a*^ (cm^2^)	*I*_p,f_^b^ (mA cm^−2^)	*E*_onset_^b^ (V)	*I*_CA_^b^ (mA cm^−2^)	*R*_ct_^b^ (Ω)
Pd/C	6.95	27.07	−0.33	4.03	35.19
Pd@rGO/In_2_O_3_	37.12	110.87	−0.47	20.96	19.21
PdAg@rGO/In_2_O_3_	40.39	147.44	−0.51	31.81	16.05
PdPb@rGO/In_2_O_3_	43.47	194.36	−0.52	44.89	9.52
PdPbAg@rGO/In_2_O_3_	47.39	213.89	−0.56	64.79	8.65

The electrocatalytic performance of various catalysts were preliminarily investigated by CV at the scanning rate of 50 mV s^−1^ in 1.0 M KOH and 0.5 M EG solution. The CV curves of EGOR (Fig. 5(B)) contains two clear oxidation peaks in both the front and back scans, the forward scan is the result of the oxidation of chemisorbed EG molecules, while the other one in the backward scan may be attributed to the further oxidation of new intermediates formed in the previous scan.^41^ As seen, the trimetallic PdPbAg@rGO/In_2_O_3_ catalyst displays the highest electrocatalytic activity among all the catalysts, and the forward current density (*I*_p,f_) is 213.89 mA cm^−2^, 7.90, 1.93, 1.45 and 1.10 times higher than those of commercial Pd/C (27.07 mA cm^−2^), Pd@rGO/In_2_O_3_ (110.87 mA cm^−2^), PdAg@rGO/In_2_O_3_ (147.44 mA cm^−2^) and PdPb@rGO/In_2_O_3_ (194.36 mA cm^−2^), respectively. And the *E*_onset_ of PdPbAg@rGO/In_2_O_3_ (- 0.56 V) is lower than those of PdPb@rGO/In_2_O_3_ (- 0.52 V), PdAg@rGO/In_2_O_3_ (- 0.51 V), Pd@rGO/In_2_O_3_ (- 0.47 V) and Pd/C (- 0.33 V). The above results fully demonstrate that the PdPbAg@rGO/In_2_O_3_ catalyst has the highest catalytic activity for electro-oxidation, the lowest overpotential and activation energy. This is due to the strong electronic effect of the PdPbAg nanoalloy, which increases the electron density of the Pd-d band. Because the adsorption of carbon-based intermediates is alleviated, the connection between Pd and poisoning species is weakened. Thus, its anti-toxicity is enhanced and the electrocatalytic oxidation of EG is promoted.^36,42^ Meanwhile, a large amount of oxide species are adsorbed on the catalyst surface. This accelerates the oxidative removal of toxic intermediates, exposing more EGOR active centers.^42,43^

**Fig. 6:**
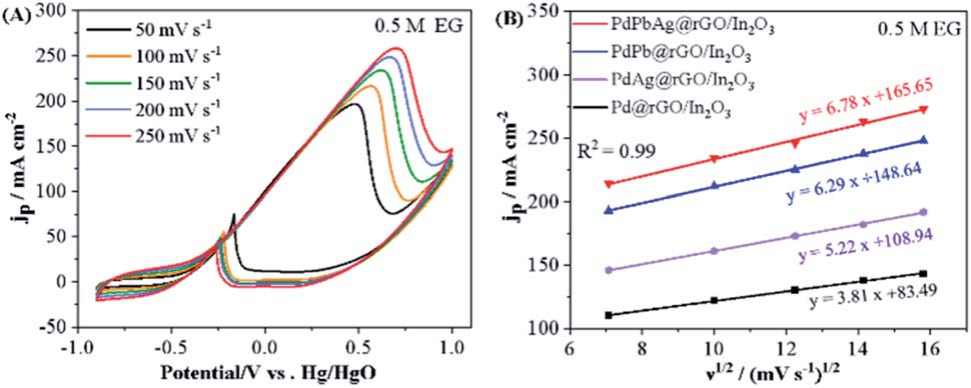
(A) EGOR curves of PdPbAg@rGO/In_2_O_3_ in 1 M KOH + 0.5 M EG solution at various scan rates. (B) curves of peak current density (*j*_P_) of four different catalysts and the square root of the scanning rate (*ν*^1/2^).

The kinetics of PdPbAg@rGO/In_2_O_3_ catalyst toward EG oxidation reaction was investigated. As shown in Fig. 6(A), the peak current density (*j*_P_) increases when the potential scan rate increases from 50 mV s^−1^ to 250 mV s^−1^. In the meantime, the peak potential (*ν*) of the CV curves shifts continuously, indicating that the electrocatalytic oxidation of EG on the PdPbAg@rGO/In_2_O_3_ catalyst electrode is an irreversible electrode process.^44^ Furthermore, the linear relationship between the square root scan velocity (*ν*^1/2^) and the forward peak current density is shown in Fig. 6(B). The linear correlation diffusion factor (*R*^2^) of the diffusion is 0.99, demonstrating that EGOR on PdPbAg@rGO/In_2_O_3_ is a diffusion-controlled irreversible electrode process.^45^ Higher slope values indicate better electro-oxidative kinetics. It is not difficult to find that among all the catalysts, the PdPbAg@rGO/In_2_O_3_ trimetallic catalyst has the best EGOR kinetics.

**Fig. 7:**
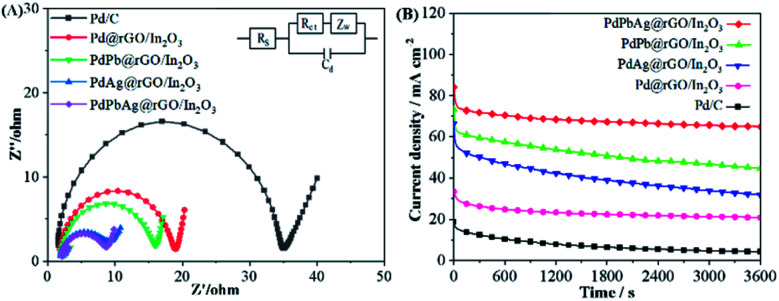
Nyquist plots of the five catalysts in (A) 1.0 M KOH + 0.5 M EG. Chronoamperometric curves of the five catalysts in (B) 1.0 M KOH + 0.5 M EG for 3600 s.

The kinetics of charge transfer and diffusion at the electrode/electrolyte interface were analyzed by electrochemical impedance spectroscopy (EIS).^21^Fig. 7(A) is the Nyquist diagram and an equivalent circuit diagram of the electrocatalyst reaction process analyzed by electrochemical impedance spectroscopy. The charge transfer resistance of the catalyst was evaluated as a semicircular diameter. Compared with PdPb@rGO/In_2_O_3_ (9.52 Ω), PdAg@rGO/In_2_O_3_ (16.05 Ω), Pd@rGO/In_2_O_3_ (19.21 Ω) and Pd/C (35.19 Ω), PdPbAg@rGO/In_2_O_3_ (8.65 Ω) has minimal impefance. It is well known that a smaller *R*_ct_ value is beneficial to the charge transfer from electrode to fuel, reducing the activation barrier of fuel oxidation and electrode reaction overpotential.^44^ Therefore, PdPbAg@rGO/In_2_O_3_ has the most superior charge transport characteristics and the largest reaction driving force.^46^

To investigate the long-term stability of the electrocatalysts, we conducted the analysis by the chronoamperometry (CA) test operating in 1.0 M KOH + 0.5 M EG for 3600 s at - 0.1 V, as shown in Fig. 7(B). The current densities of the five catalysts drop rapidly in the initial stage due to the accumulation of toxic intermediates, resulting in a severe drop in catalytic activity.^41^ The current density then decreases slowly until it reaches a relatively stable state. It can be seen that among the electrocatalysts, PdPbAg@rGO/In_2_O_3_ always maintains the highest EG electrooxidation activity. The results show that the PdPbAg@rGO/In_2_O_3_ has the best catalytic durability for EG. The retained current density of PdPbAg@rGO/In_2_O_3_ (64.79 mA cm^−2^) is superior to that of Pd/C (4.03 mA cm^−2^, 20.15%), Pd@rGO/In_2_O_3_ (20.96 mA cm^−2^), PdAg@rGO/In_2_O_3_ (31.81 mA cm^−2^) and PdPb@rGO/In_2_O_3_ (44.89 mA cm^−2^). Among them, compared with Pd/C (20.15%), the current retention ratio of the thrimetallic electrocatalyst (78.15%). This is due to the fact that PdPbAg@rGO/In_2_O_3_ catalyst has sufficient metal oxides and the highest effective activation specific surface area.

To further clarify the excellent electrocatalytic activity of PdPbAg@rGO/In_2_O_3_, we propose the electrocatalytic oxidation mechanism of EG and the removal process of CO_(ads)_ on Pd sites, as the following steps:^36,42,43^

1 (CH_2_–OH)_2_ → (CH_2_–OH)_2(ads)_

2 OH^−^ → OH^−^_(ads)_

3 (CH_2_–OH)_2(ads)_ + 4OH^−^_(ads)_ → (CHO)(CHO) + 4H_2_O + 4e^−^

4 (CHO)(CHO) + 6OH^−^_(ads)_ → (COO^−^)_2_ + 4H_2_O + 4e^−^

5 (COO^−^)_2_ + 4OH^−^_(ads)_ → 2CO_3_^2−^ + 2H_2_O + 2e^−^

6 2Pd + (CH_2_–OH)_2_ + 6OH^−^ → 2Pd–CO_(ads)_ + 6H_2_O + 6e^−^

7 Pb + OH^−^ → Pb–OH_(ads)_ + e^−^

8 PbO + OH^−^ → PbO–OH_(ads)_ + e^−^

9 Pd–CO_(ads)_ + Pb–OH_(ads)_ + 3OH^−^ → Pd + Pb + CO_3_^2−^ + 2H_2_O + e^−^

10 Pd–CO_(ads)_ + PbO–OH_(ads)_ + 3OH^−^ → Pd + PbO + CO_3_^2−^ + 2H_2_O + e^−^

From the above reaction mechanism, it can be found that the more active sites of the multi-metallic catalyst, the more oxygen-containing species adsorbed, and the more conducive to the electrocatalytic oxidation of EGOR. Compared with Pd/C and bimetallic catalysts, PdPbAg@rGO/In_2_O_3_ trimetallic catalyst contains more active sites. The strong synergistic effect of Pd–Pb–Ag alloy NPs can adsorb more OH^−^_(ads)_ and (CH_2_OH)_2(ads)_, thus exhibiting the best electro-oxidative performance. From this, we can infer that the intermediate CO_(ads)_ on the electrode surface is easily converted to CO_2_.^47^ On the one hand, the toxic intermediates in the active site of Pd are eliminated in time, so that fresh Pd can further absorb and oxidize EG molecules. On the other hand, Pb can form OH functional groups on the catalyst surface. This promotes the oxidation of carbonaceous species, thereby improving the oxidative stability of EG. Meanwhile, the high electronic conductivity of the supported rGO/In_2_O_3_ is beneficial for metal loading and electrocatalysis. From the above analysis, it can be further proved that PdPbAg@rGO/In_2_O_3_ has faster electron transfer and better catalysis for the electro-oxidation of EG.

## Conclusions

A novel composite GO/In_2_O_3_ was prepared by hydrothermal and calcination method, and the well-dispersed Pd–Pb–Ag alloy NPs were immobilized on GO/In_2_O_3_ by impregnation reduction method. Compared with PdPb@rGO/In_2_O_3_, PdAg@rGO/In_2_O_3_, Pd@rGO/In_2_O_3_ and Pd/C, the finally synthesized anode catalyst PdPbAg@rGO/In_2_O_3_ has the highest catalytic activity, the lowest *E*_onset_ (−0.56 V), the smallest impedance for EG (8.65 Ω), and better resistance to toxicity. Among them, the *I*_p,f_ of the ternary electrocatalyst PdPbAg@rGO/In_2_O_3_ (213.89 mA cm^−2^) for the electrooxidation of EG is 7.89 times higher than that of Pd/C (27.07 mA cm^−2^). After 3600 s, PdPbAg@rGO/In_2_O_3_ still maintained the highest catalytic activity and durability, the retained current density of PdPbAg@rGO/In_2_O_3_ (64.79 mA cm^−2^) reaches 78.15% of the initial value, which is superior to that of Pd/C (4.03 mA cm^−2^, 20.15%). This outstanding electrocatalytic performance is related to the surface modification of the support rGO/In_2_O_3_. This structure improves the specific surface area and electron transfer rate. And its good electrical conductivity provides abundant active sites for the Pd–Pb–Ag alloy NPs. Meanwhile, the strong synergistic effect of the formed Pd–Pb–Ag alloy NPs can significantly enhance the adsorption of oxygen-containing species on the Pd surface. Thereby accelerating the oxidation of intermediate carbon species on the Pd surface and the removal of adsorbed CO species. The design of this composite and its efficient catalytic performance will provide important ideas for the development of a wide range of energy catalysts.

## Author contributions

Zhirui Wu: conceptualization, experimental, investigation, formal analysis, writing-original draft and writing-review & editing. Yuting Zhong: conceptualization and investigation. Zhiguo Wang: resources and project administration. Ling Li: project administration and resources. Xiaoguang Liu: supervision and writing-review & editing.

## Conflicts of interest

There are no conflicts to declare.

## Supplementary Material
